# ﻿Multilocus phylogeny and species delimitation suggest synonymies of two *Lucanus* Scopoli, 1763 (Coleoptera, Lucanidae) species names

**DOI:** 10.3897/zookeys.1135.89257

**Published:** 2022-12-14

**Authors:** Li Yang Zhou, Zhi Hong Zhan, Xue Li Zhu, Xia Wan

**Affiliations:** 1 Department of Ecology, School of Resources and Engineering, Anhui University, 111 Jiulong Rd., Hefei 230601, China; 2 Anhui Province Key Laboratory of Wetland Ecosystem Protection and Restoration, Anhui University, 111 Jiulong Rd., Hefei, 230601, China; 3 Department of Entomology, University of Wisconsin Madison, WI 53706, USA

**Keywords:** genetic distance, Lucanidae, morphology, new synonymy, phylogenetic analysis, species delimitation

## Abstract

Phylogenetic relationsships of four nominal *Lucanus* Scopoli, 1763 species, *L.swinhoei* Parry, 1874, *L.continentalis* Zilioli, 1998, *L.liuyei* Huang & Chen, 2010, and *L.wuyishanensis* Schenk, 1999, are assessed based on mitochondrial (16S rDNA, COI) and nuclear (28S rDNA, Wingless) genes. The genetic distance is 0.0072 between *L.swinhoei* and *L.continentalis*, and 0.0094 between *L.wuyishanensis* and *L.liuyei*. Three species-delimitation approaches (ABGD, PTP, and GMYC) consistently showed *L.swinhoei* + *L.continentalis* and *L.wuyishanensis* + *L.liuyei* as two MOTUs. A new synonymy, *L.liuyei* = *L.wuyishanensis*, is proposed. Synonymy of *L.swinhoei* over *L.continentalis* is confirmed.

## ﻿Introduction

Morphological evidence suggests that the evolution and differences of the mandible of stag-beetles are closely related to environmental heterogeneity (Huang and Lin 2010; Gotoh et al. 2011). Molecular phylogeny also provides evidence for the intraspecific morphological complexity due to environmental heterogeneity in *Lucanus* (Zhou et al. 2019; Ying et al. 2021; Yuan et al. 2021).

The genus *Lucanus* Scopoli, 1763 is recognized as the most typical representative of Lucanidae, and *Lucanus* species (and subspecies) are especially abundant in eastern regions of Asia (including China, India, Laos, Vietnam, and Myanmar), with the majority inhabiting southern China (Wan 2007; Fujita 2010; Huang and Chen 2010; Lin 2017; Chen et al 2020). Hilly topography below 2000 m dominates the central and eastern regions of south China, with many low mountains and valley basins extending from northeast to southwest (Zhou et al. 2006). This unique topography may have hindered gene exchange between species and facilitated population differentiation (Qiu et al. 2011; Zhao et al. 2012; Li et al. 2019). Secondly, sexual dimorphism, male polymorphism, and color pattern polymorphism are significant in *Lucanus*. Due to the above reasons, this genus is phenotypically rich at the intraspecific level, resulting in some taxonomic confusion. Phylogenetic analysis using molecular markers such as mitochondrial and nuclear genes can clarify many morphology-based species taxonomic positions.

**Figure 1.**
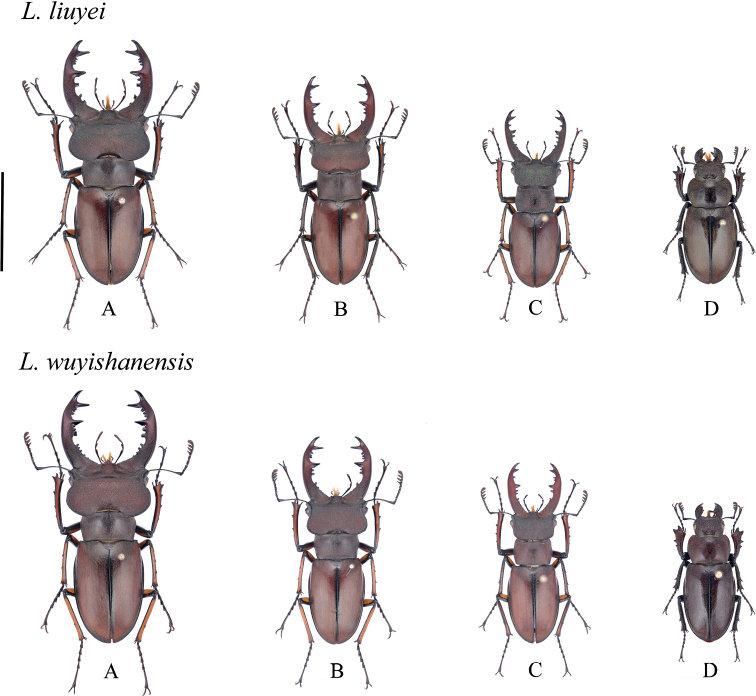
Habitus of *L.wuyishanensis* and *L.liuyei* in dorsal view **A** major male **B** medium male **C** minor male **D** female. All to scale; scale bar: 20.0 mm.

*Lucanuswuyishanensis* Schenk, 1999 and *Lucanusliuyei* Huang & Chen, 2010 are typical representatives of *Lucanus*. All collecting data indicate that *L.wuyishanensis* is mainly distributed in southeast China (Zhejiang, Jiangxi, Fujian). Its allied species, *L.liuyei* in south-central China (Guangxi, Guizhou, Hunan), is morphologically similar to *L.wuyishanensis* but has a different geographic distribution. Huang (2006) erroneously treated *L.wuyishanensis* as a synonym of *Lucanusklapperichi*; however, examination of the male genitalia proved that this species should be treated as distinct (Huang and Chen 2010). Similarly, Wan (2007) also believed that the lateral ridges of the head and the major inner tooth are quite different from those of *L.klapperichi*. Additionally, Huang and Chen (2013) observed that *L.liuyei* from Guangxi is closer to *L.wuyishanensis*, from the border area of Jiangxi, Zhejiang, and Fujian, in terms of external morphology, and classified it as a subspecies of *L.liuyei* by comparing their male and female genitalia. However, the specimens collected in Guangxi were identified by Fujita (2010) as *L.wuyishanensis* based on morphological characters. In our opinion, the differences between the two species are insignificant and hardly any diagnostic attribute was found to distinguish them except for existing collecting data showing distribution areas and body size ranges (Fig. 1).

**Figure 2.**
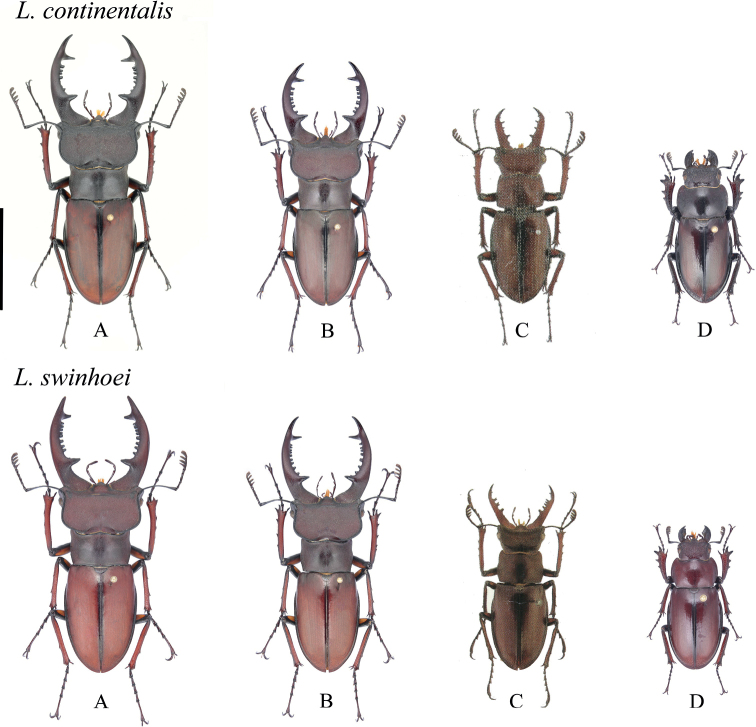
Habitus of *L.continentalis* and *L.swinhoei* in dorsal view **A** major male **B** medium male **C** minor male (see Huang and Chen 2010: pl. 35 figs 34–5, 9) **D** female. Scale bar: 20.0 mm.

In addition, the taxonomic relationship between *Lucanusswinhoei* Parry, 1874 and *Lucanuscontinentalis* Zilioli, 1998 has long been controversial. Zilioli (1998) reported that *L.continentalis* is a subspecies of *L.swinhoei*; however, Wan (2007) considered *L.continentalis* as a synonym of *L.swinhoei* based on examination of a series of specimens. Huang and Chen (2010) compared the inner teeth, the margin of the basal part of the mandible, the labrum, and the geographical distribution of the two species and concluded that they are separate species, with the former mainly distributed in southeastern China and the latter only in Taiwan. Later, Huang and Chen (2017) relegated *L.continentalis* to a subspecies of *L.swinhoei*. So far, the taxonomic positions of *L.swinhoei* have been listed as distinct species, subspecies, or synonyms despite a lack of abundant data to support these modifications (Fig. 2).

This study assesses the taxonomic relationships between the species pairs *L.continentalis* and *L.swinhoei*, and *L.wuyishanensis* and *L.liuyei*, by using multi-locus data, revisiting for the first time the relationships among these four species from a molecular phylogenetic perspective.

## ﻿Materials and methods

### ﻿Sample collection, handling, and storage

All specimens of *Lucanus* were netted or light-trap collected for this study and store in ethanol, including 54 samples of the ingroup (21 *L.wuyishanensis* collected from Zhejiang, Fujian, Jiangxi province; 17 *L.liuyei* from Guangxi, Guizhou, Hunan province; five *L.swinhoei* from Taiwan; 11 *L.continentalis* from Fujian and Zhejiang province), and 10 samples of the outgroup (one each of *L.parryi* Boileau, 1899, *L simithii* Parry, 1862, *L.fryi* Boileau, 1911, *L.klapperichi* Bomans, 1989, and six *L.fujianensis* Schenk, 2008). Voucher specimens and their extracted genomic DNA are deposited in the research collection at the Museum of Anhui University, China. (Suppl. material 1).

**Figure 3.**
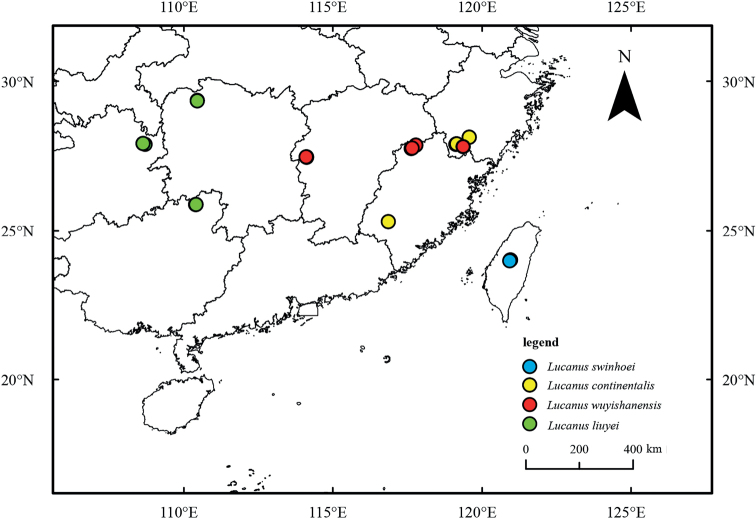
Sample collection sites for this study.

The map with collection localities was generated using ArcGIS v. 10.3 (http://www.esri.com/sofware/arcgis) based on the geospatial data from the National Geomatics Center of China (Fig. 3). Photographs of the habitus was taken in .jpg format using a Canon 5D Mark IV with Canon 100 mm f/2.8 macro lens and a twin flash (Figs 1, 2).

### ﻿DNA extraction, amplification, and sequencing

The specimens were preserved in 99.7% ethanol at −20 °C. Total genomic DNA was extracted from a small portion of the muscle using DNeasy Blood and Tissue Extraction kit according to the manufacturer’s recommendations. The primers used to amplify 28S rDNA and Wingless were adapted from Abouheif and Wray (2002), Ward and Downie (2005), Monaghan et al. (2007), and Wild and Maddison (2008). For COI and 16S rDNA, primers were specifically designed in this study (Table 1).

**Table 1.** Summary of paired PCR primers in the present study.

Gene	Primer	Sequence (5´–3´)	References
COI	LuCOIF1	ATAATCATTGCTGTTCCAAC	Present study
	LuCOIR1	TATCTATGTTCAGCRGGRGGT	Present study
16S rDNA	Lu16SF1	CTCGAATTTTRGAGGGC	Present study
	Lu16SR1	AATCCAACATCGAGGTC	Present study
28S rDNA	28SDD	GGGACCCGTCTTGAAACAC	Monaghan et al. 2007
	28SFF	TTACACACTCCTTAGCGGAT	Monaghan et al. 2007
Wing-less	Wg550F	ATGCGTCAGGARTGYAARTGYCAYGGYATGTC	Wild and Maddison 2008
	WgAbRZ	CACTTNACYTCRCARCACCARTG	Wild and Maddison 2008
	Wg578F WgAbR	TGCACNGTGAARACYTGCTGGATG ACYTCGCAGCACCARTGGAA	Ward and Downie 2005 Abouheif and Wray 2002

PCR amplification reactions for the three loci (COI, 16S rDNA, 28S rDNA) were performed in a 25 μL volume containing 1 μL of each primer (forward and reverse) at 10 μM, 2 μL of template DNA solution, 12.5 μL of 2× EasyTaq SuperMix (+ dye), and 8.5 μL of sterile double-distilled water to make up the final volume of 25 μL. WG was amplified by nested PCR, first PCR containing 1 μL of each primer (forward and reverse) at 10 μM, 1 μL of template DNA solution, 7.5 μL of 2× EasyTaq SuperMix (+ dye), and 4.5 μL of sterile double-distilled water, and finally use the 1 μL first amplification product as a template, including 1 μL of each primer (forward and reverse) at 10 μM, 12.5 μL of 2× EasyTaq SuperMix (+ dye), and 9.5 μL of sterile double-distilled water. The polymerase chain reaction amplifications were performed under the following conditions: initial denaturation at 94 °C for 2 min, followed by 35–37 cycles of denaturation at 94 °C for 40 seconds, annealing at 52–60 °C for 50 seconds, and elongation at 70 °C for 1 min, and then a final extension step at 72 °C for 7 min, stored at 4 °C at room temperature. Amplifications were purified using Template DNA Amplify Kit (Ensure Biologicals).

Sequencing was performed using the ABI PRISM BigDye Terminator v. 3.1 Cycle Sequencing Kit (Life Technologies, USA), and cycle sequencing reactions were performed on ABI PRISM 3730xl automated sequencers (Life Technologies, USA) at Sangon Biotech Company, China. All sequences generated in this study were submitted to GenBank under accession numbers (Suppl. material 1).

### ﻿Sequence alignment, genetic distances, and phylogenetic analyses

Sequences of forward and reverse strands were assembled using GENEIOUS PRIME 2019.1.1 (https://www.geneious.com) and then aligned using MEGA 11. Genetic divergences among taxa were estimated using MEGA 11 (Kumar et al. 2018) via K2P-distance. The COI gene of *Lucanus* was assembled for genetic distance analyses. Finally, we concatenated alignments using PHYLOSUITE 1.2.1 (Zhang et al. 2020). The concatenated dataset was partitioned according to the Akaike Information Criterion (AIC) with PARTITIONFINDER 2.1.1 (Lanfear et al. 2017) for phylogenetic analyses.

Phylogenetic inferences were conducted using four gene markers based on maximum likelihood inference (**ML**), and Bayesian inference (**BI**). The BI tree was implemented in MRBAYES 3.2.6 (Ronquist et al. 2012). PARTITIONFINDER 2.1.1 was used to determine the best-fit models. Bayesian inference was conducted using MRBAYES 3.2.6 with two simultaneous runs of 5 × 10^7^ generations. Samples were drawn every 1,000 Markov Chain Monte Carlo (MCMC) step. The average standard deviation of split frequencies should be less than 0.01, with the initial 25% of trees discarded as burn-in. ML analyses were performed using IQ-TREE webserver (Trifinopoulos and Lam-Tung 2016). The “Auto” option was set under optimal evolutionary models, and the phylogenetic trees were constructed using an ultrafast bootstrap approximation approach with 10,000 replicates. Phylogenetic trees were visualized and edited in FIGTREE 1.4.3 (http://beast.bio.ed.ac.uk/figtree).

### ﻿Species delimitation

When defining species relationships by using molecular-set data, there are a variety of analytical approaches available. The Automatic Barcode Gap Discovery (**ABGD**) analysis was performed in this study for COI using a web interface (https://bioinfo.mnhn.fr/abi/public/abgd/abgdweb.html), which detects a gap in divergence distribution, which corresponds to differences between intraspecific and interspecific distances. When the gap exists, the process works well for species delimitation (Puillandre et al. 2012). An unrooted ML tree was generated in IQ-TREE webserver under the auto options. Poisson Tree Processes (**PTP**) was performed on a single, unrooted tree in the bPTP server (https://species.h-its.org/). A total of 5 × 10^5^ generations were run with the first 10% as burn-in (Zhang et al. 2013). General Mixed Yule Coalescent model (**GMYC**) analysis was conducted using BEAST 2.6.0 under a relaxed clock Exponential mode and ESS values assessed convergence. A burn-in with 25% was set to obtain an optimal consensus tree. Delimitation approach in the software R with the package ‘splits’ (available at http://r-forge.r-project.org/projects/splits) using the single-threshold method (Pons et al. 2006; Monaghan et al. 2009).

### ﻿Abbreviations

**ABGD** Automatic Barcode Gap Discovery;

**BBP** Bayesian posterior probability;

**BI** Bayesian inference;

**GMYC** General Mixed Yule Coalescent;

**K2P** distance, Kimura 2-parameter distance;

**ML** maximum likelihood;

**MBL** maximum likelihood bootstrap;

**MOTU** molecular operational taxonomic unit;

**PCR** Polymerase Chain Reaction;

**PTP** Poisson Tree Processes.

## ﻿Results

### ﻿Morphological comparison

External morphological characteristics of two clades are compared in the table (Suppl. material 2). *Lucanusswinhoei* and *L.continentalis* differ slightly in body size, maintaining an overall range of 27–58 mm. Major males display the following features: mandibles strongly incurved at basal 1/3 and at apex; apical teeth bifurcated, upper branch teeth usually larger or equal in size with lower branch teeth; major mandibular tooth located at basal 1/3, triangular, proceeded by more than five inner small teeth. Medium-sized males have the following features: mandibles strongly incurved at basal 1/3, usually straight at apex; apical teeth bifurcated, upper branch teeth usually equal to or smaller than lower branch teeth; major mandibular tooth located at basal 1/3, triangular, proceeded by four or five inner small teeth. Minor males indicate the following features: mandibles weakly incurved at basal 1/3, straight at apex; apical teeth bifurcated, upper branch teeth usually equal to or smaller than lower branch teeth; major mandibular tooth located at basal 1/3, triangular, proceeded by fewer than four inner small teeth.

*Lucanuswuyishanensis* and *L.liuyei* differ slightly in body size, maintaining an overall range of 28–53 mm. Major males indicate the following features: mandible weekly incurved at basal 1/3, straight at the middle then strongly incurved at apical 1/4; the major inner mandibular tooth located 2/3 from the apical mandibular fork, sharp, triangular protruding forward and inflated on both sides, four separated small inner mandibular teeth attached below the major inner mandibular tooth, four or five unclear, minor inner mandibular teeth continuously located along the midlength of basal mandibles; four small inner mandibular teeth densely distributed between the major tooth and the apical fork. Medium-sized males indicate the following features: major inner mandibular tooth somewhat triangular, weekly inflated on both sides; more than four unclear, minor inner mandibular teeth continuously located along the midlength of basal mandibles, and more than four small inner mandibular teeth densely distributed between the major tooth and the apical fork. Minor males indicate the following features: major inner mandibular tooth weekly developed, single-point and not triangular; less than two separated small inner mandibular teeth attached below the major inner mandibular tooth; more than three unclear, minor inner mandibular teeth continuously located on 1/2 of basal mandible; more than two small inner mandibular teeth densely distributed between the major tooth and the apical fork.

### ﻿Phylogenetic analyses

**Figure 4.**
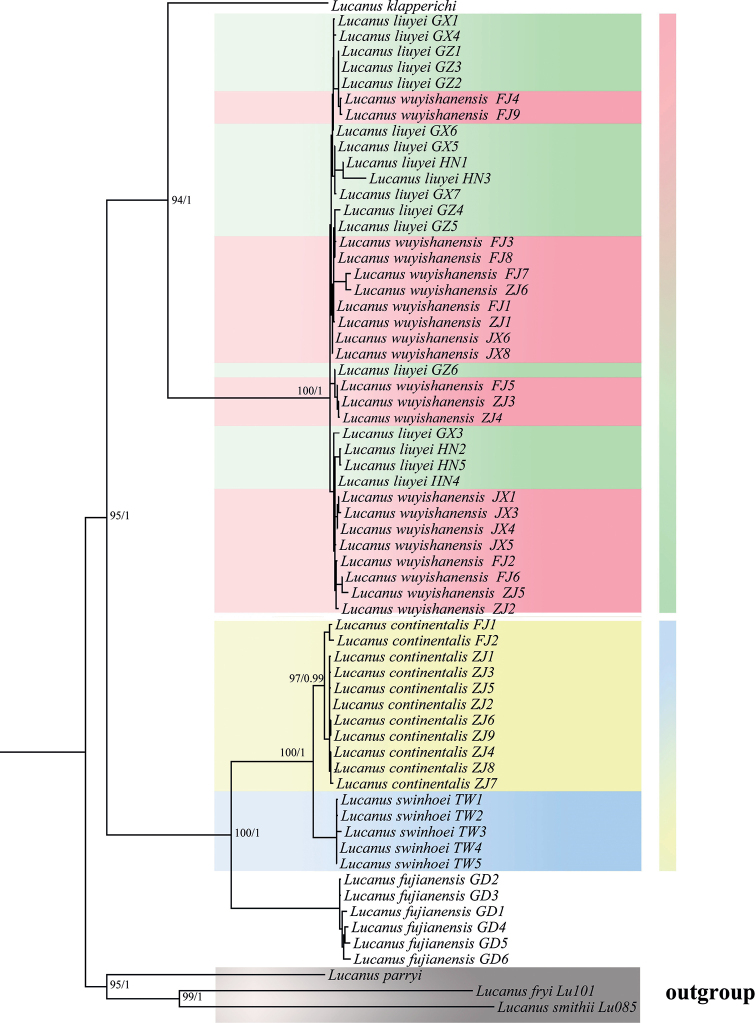
Phylogenetic inferences based on four genes (COI, 16S rDNA, Wingless, and 28S rDNA) by maximum-likelihood inference (MLI) and Bayesian inference (BI) with posterior probability. Both posterior probabilities of MLI (above/left of branch) and bootstrapping values of BI (below/right of branch) are shown at nodes.

A concentrated matrix with 2489 aligned positions for data was obtained comprising COI, 16S rDNA, 28S rDNA, and Wingless genes. The phylogenetic analyses using both BI and ML inferences recovered overall a consistent topology (Fig. 4). As outgroups, *L.smithii*, *L.fryi*, and *L.parryi* were separated from other species with high support, forming an independent clade. In addition, the clade *L.klapperichi* was sister to the clade (*L.liuyei* + *L.wuyishanensis*) (BPP = 1, MLB = 94). The clade *L.fujianensis* was sister to the clade (*L.swinhoei* + *L.continentalis*) (BPP = 1, MLB = 100). Nested structures of *L.liuyei* and *L.wuyishanensis* occur in all geographic populations.

### ﻿Genetic distance

Genetic distances (K2P-distances) were calculated for all taxa using COI genes (Table 2). The results showed that the average genetic distance between *L.wuyishanensis* and *L.liuyei* populations in each collection area was a low mean range (0.0067–0.0110) (Suppl. material 3) and mean genetic distance of 0.0094 in all taxa (Table 2); the mean genetic distance between *L.swinhoei* and *L.continentalis* was 0.0072 (Table 2). The numbers were lower than the minimum mean genetic distances of 0.1592 among interspecific taxa and far less than the mean genetic distance of 0.2090 between interspecies of *Lucanus* (Lin 2017).

**Table 2.** The mean genetic distances among studied species (K2P-distances).

	*L.liuyei*	*L.wuyishanensis*	*L.continentalis*	*L.swinhoei*	*L.fujianensis*	*L.klapperichi*	*L.fryi*	*L.smithii*	*L.parryi*
*L.liuyei*									
*L.wuyishanensis*	0.0094								
*L.continentalis*	0.1949	0.1924							
*L.swinhoei*	0.1906	0.1872	0.0072						
*L.fujianensis*	0.2131	0.2142	0.1617	0.1592					
*L.klapperichi*	0.1946	0.1977	0.1925	0.1872	0.1929				
*L.fryi*	0.2067	0.2086	0.2321	0.2280	0.1885	0.1955			
*L.smithii*	0.2246	0.2245	0.2150	0.2176	0.2230	0.2434	0.1962		
*L.parryi*	0.2280	0.2306	0.2193	0.2164	0.2054	0.1840	0.1774	0.1987	

### ﻿Species delimitation

**Figure 5.**
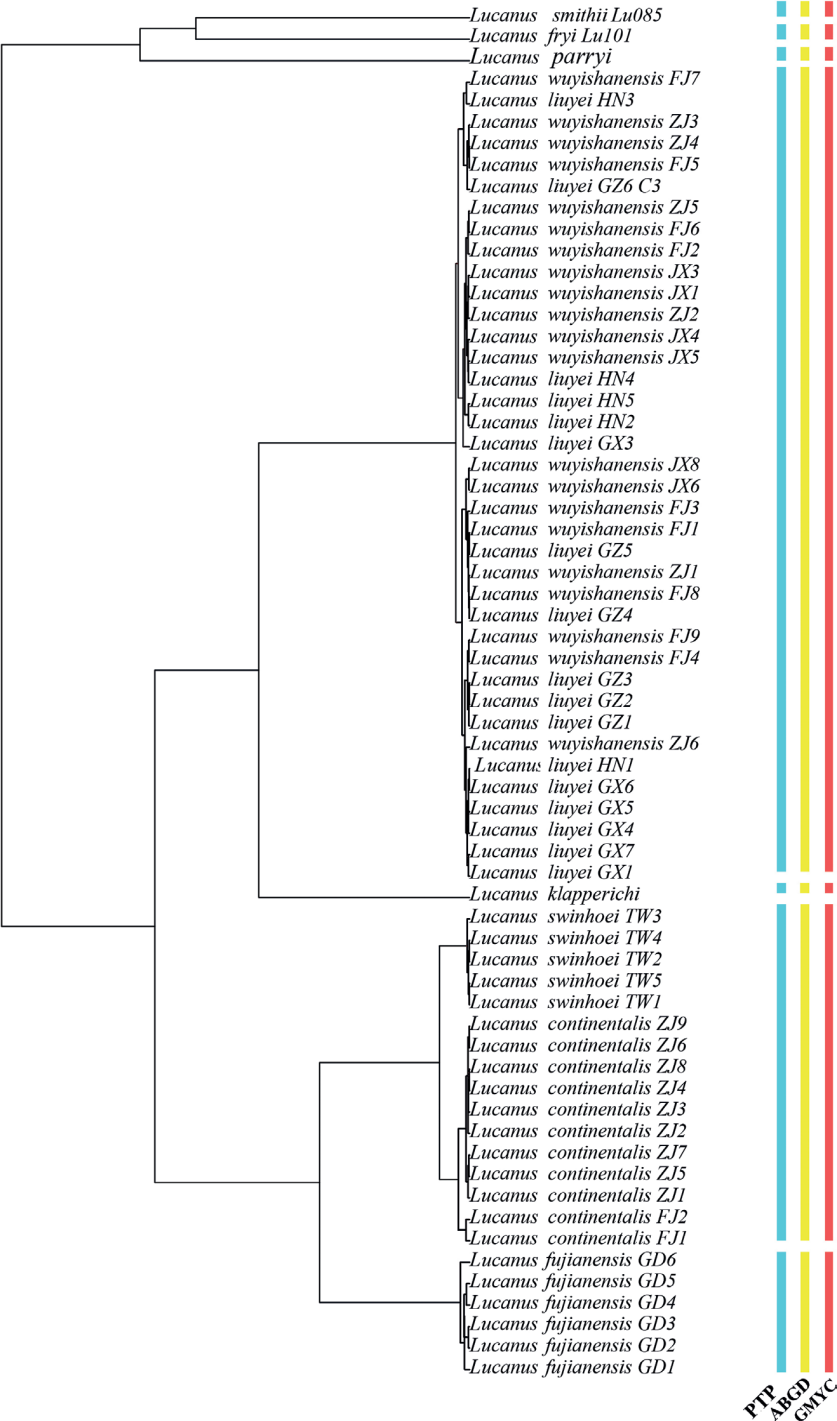
Delimitation of the studied *Lucanus* species based on COI. Columns are taxonomic identification based on three molecular delimitation methods: ABGD, PTP, and GMYC. The phylogenetic tree is based on the GMYC analysis.

Species delimitation is shown in Fig. 5. Analysis of COI gene by all methods (ABGD, PTP, and GMYC) resulted in two molecular MOTUs, *L.wuyishanensis* + *L.liuyei* and *L.swinhoei* + *L.continentalis* (Fig. 5). For the concatenated dataset, all three methods suggested that *L.wuyishanensis* + *L.liuyei* were one MOTU, whereas GMYC divided *L.swinhoei* and *L.continentalis* into two MOTUs.

### ﻿Taxonomic account

#### ﻿ Lucanus swinhoei

Taxon classificationAnimaliaColeopteraLucanidae

Parry, 1897

36031733-8556-5357-9A7C-BEB8D45A3AED

Lucanus swinhoei Parry, 1874: 370.
Lucanus continentalis Zilioli, 1998: 145, synonymy by Wan (2007).

##### Material examined.

China • 1 male; Zhejiang Province, Yunhe County; 18 Jul. 2019; ZH Zhan leg. • 3 males; same locality as for preceding; 5 Jul. 2021 • 3 males; Zhejiang Province, Baishanzu County; 23 Jul. 2015 • 2 males; Zhejiang Province, Longquan County; 6 Jul. 2019 • 1 male; Fujian Province, Shanghang County; 12 Jul. 2021; LY Zhou leg. 1 male; same locality as for preceding; 22 Jul. 2011; Q Zhang and YY Cao leg. • 2 males; Taiwan Island, Nantou County; 12 Jun. 2019; JZ Lin leg. • 2 males; same locality as for preceding; 6 Jun. 2020.

##### Diagnosis.

Males of *L.swinhoei* could be distinguished from related species by following characters: 1) mandibles incurved at basal 1/3, straight along the midlength and incurved apically; 2) apical teeth bifurcated; major mandibular tooth located at basal 1/3, triangular, not flat on both sides; 3) elytra metallic luster at disc and along the suture, reddish to brownish; less punctate and without any yellowish setae. The females of most species in *Lucanus* are not easy to distinguish due to their significant similarities in morphology. Typical female of *L.swinhoei* could be identified by the following subtle differences: elytra without a marked pubescence, metasternum not densely hairy and the canthi not markedly outside of the eyes.

##### Distribution.

China (Zhejiang, Fujian, Taiwan Island).

#### ﻿ Lucanus wuyishanensis

Taxon classificationAnimaliaColeopteraLucanidae

Schenk, 1999

F9E934C9-9AFE-5526-9C6D-FA07A683D198

Lucanus wuyishanensis Schenk, 1999: 114.
Lucanus liuyei Huang & Chen, 2010: 93–94, syn. nov.

##### Material examined.

China • 2 males; Jiangxi Province, Pingxiang County; 15 Jun. 2017; ZH Zhan leg. • 4 males; Jiangxi Province, same locality as for preceding; 03 Jun. 2021; Q Qi leg. • 6 males; Zhejiang Province, Mount Longquanshan; 18 July. 2019. • 1 male; Fujian Province, Mount Wuyishan; 18 Jul. 2011. • 2 males; Fujian Province, Mount Wuyishan; 14 Jul. 2011; Q Zhang and YY Cao leg. • 4 males; Fujian Province, same locality as for preceding; 12 Jun. 2020; ZH Zhan leg. • 2 males; Fujian Province, same locality as for preceding; 20 Jun. 2021; ZL Zhou leg. • 1 male; Guangxi Province, Mount Maoershans; 20 Jul. 2011. • 3 males; same locality as for preceding; 20 Jul. 2017; Q Qi leg. • 2 male, 1 female; same locality as for preceding; 20 Jul. 2021; ZH Zhan leg. • 2 male, 2 female; Guizhou Province, Mount Fanjingshan; 20 Jun. 2017; ZH Zhan leg. • 2 males; same locality as for preceding; 08 Jul. 2015; LX Zhu leg. • 1 male; Hunan Province, Zhangjiajie County; 20 Jun. 2015 • 4 males; same locality as for preceding; 21 May 2019; ZH Zhan leg.

##### Diagnosis.

Males of *L.wuyishanensis* could be distinguished from related species by the following characters: 1) mandibles weekly incurved at basal 1/3, straight extending to the mid-length and strongly incurved at 1/4 anteriorly; 2) two separated, small, inner mandibular teeth attached below the major inner mandibular tooth; 2–4 small inner mandibular teeth densely distributed between the major tooth and the apical fork; 3) elytra reddish to brownish, usually bicolored with head and pronotum; oval, widest at the apical 1/4, strongly narrow at basal. Females of *L.wuyishanensis* are also similar in appearance to those of other *Lucanus* members. There are the following slight differences: dorsal surface covered with a vestiture of small and significant, yellowish-amber setae; head surface punctate heavily, mandible snout, strongly incurved anteriorly.

##### Distribution.

China (Sichuan, Guangxi, Guizhou, Fujian, Hunan, Jiangxi).

## ﻿Discussion

Phylogenetic inferences by applying ML and BI analyses showed consistent patterns, which show that the *Lucanusklapperichi* clade is sister to the clade (*L.wuyishanensis* + *L.liuyei*) (MLB = 94%, BPP = 1). *Lucanuswuyishanensis* and *L.liuyei* collected from different provinces were all clustered in a highly supported clade. The subclade *L.swinhoei* + *L.continentalis* is nested in the same clade (MLB = 100%, BPP = 1; Fig. 4). The K2P genetic distances between *L.wuyishanensis* and *L.liuyei* were (0.0067–0.0110 mean genetic distance of 0.0094), indicating that two forms belong to one species. The K2P distance between *L.swinhoei* and *L.continentalis* (0.0072) suggests that the former most likely represents an island population of the latter, similar to the forms distributed on Hainan Island (Zhou et al. 2019). Three species-delimitation approaches (ABGD, PTP, and GMYC) based on the COI gene also consistently showed *L.swinhoei* + *L.continentalis* and *L.wuyishanensis* + *L.liuyei* as two MOTUs (Fig. 5). Based on the concatenated dataset analysis, three methods suggested *L.wuyishanensis* and *L.liuyei* were one MOTU, whereas GMYC divided *L.swinhoei* and *L.continentalis* into two MOTUs. GMYC typically over-splits species, owing to low genetic diversity across lineages and overlap of interspecific and intraspecific divergences, as well as a lack of reciprocal monophyly within sister clades (Talavera et al. 2013; Pentinsaari et al. 2016; Stokkan et al. 2018; Yuan et al. 2021).

The genus *Lucanus* is susceptible to several pressures, such as habitat selection, sexual selection, and food resources, and only occurs in wooded alpine areas above 800 m with more demanding environmental conditions and tiny ecological niches (Switala et al. 2014; Chen et al. 2020). Hilly topography with below 2000 m dominates the central and eastern regions of south China, with many low mountains and valley basins extending from the northeast to the southwest (Zhou et al. 2006). Therefore, we think that the small phenotypic differences previously examined are attributable to phenotypic divergence due to geographic and climatic variables.

All our results indicate that *L.continentalis* is a junior subjective synonym of *L.swinhoei* and that *L.liuyei* as a junior subjective synonym of *L.wuyishanensis*. It is also clear, that in case of closely related species of the genus, an integrative approach utilizing both morphological and molecular data should be used. Molecular data can provide insight into the status of the forms with weak morphological differences. It is especially important for *Lucanus* and majority of other stag beetles because molecular traits are not prone to allometric variability.

## Supplementary Material

XML Treatment for Lucanus swinhoei

XML Treatment for Lucanus wuyishanensis

## Supplementary materials

Supplementary material 1 A list of specimens’ voucher information and GenBank accession numbers used in this study Data type occurences, phylogenetic (excel document) Explanation note The table contains the name of the organism, the sample storage number, the location of the sample collection, latitude and longitude, and GenBank accession numbers. https://binary.pensoft.net/file/781699This dataset is made available under the Open Database License (http://opendatacommons.org/licenses/odbl/1.0/). The Open Database License (ODbL) is a license agreement intended to allow users to freely share, modify, and use this Dataset while maintaining this same freedom for others, provided that the original source and author(s) are credited.Author: Li Yang Zhou

Supplementary material 2 Morphological comparisons between phylogenetic clades Data type morphological (word document) https://binary.pensoft.net/file/781700This dataset is made available under the Open Database License (http://opendatacommons.org/licenses/odbl/1.0/). The Open Database License (ODbL) is a license agreement intended to allow users to freely share, modify, and use this Dataset while maintaining this same freedom for others, provided that the original source and author(s) are credited.Author: Li Yang Zhou

Supplementary material 3 The mean genetic distance among populations in each collection area (K2P-distances) Data type genetic distance (word document) Explanation note Interspecific pairwise comparison using K2P-distances based on COI among populations in each collection area. https://binary.pensoft.net/file/781701This dataset is made available under the Open Database License (http://opendatacommons.org/licenses/odbl/1.0/). The Open Database License (ODbL) is a license agreement intended to allow users to freely share, modify, and use this Dataset while maintaining this same freedom for others, provided that the original source and author(s) are credited.Author: Li Yang Zhou
