# Supplementary material for: ﻿Multilocus phylogeny and species delimitation suggest synonymies of two Lucanus Scopoli, 1763 (Coleoptera, Lucanidae) species names
Source: Zookeys. 2022 Dec 14;1135:139–55. doi: 10.3897/zookeys.1135.89257 (PMC9836572; doi:10.3897/zookeys.1135.89257)
Supplement: Supplementary material 2 — Morphological comparisons between phylogenetic clades [file zookeys-1135-139_article-89257__-s002.docx]

**Supplementary file 2: Table S2:** Morphological comparisons between phylogenetic clades

| Species | Length | Head | Mandible | Pronotum | Thorax | Elytra |
| --- | --- | --- | --- | --- | --- | --- |
| *Lucanus swinhoei*  *Lucanus continentalis* | 27–56.7mm  30.2–57.5mm | Reddish or brownish, wider than long; clearly punctate and without any yellowish setae. | **Major males** (48–55 mm): strongly incurved at basal 1/3 and at apex; Apical teeth bifurcated, upper branch teeth usually larger or equal with lower branch teeth; major mandibular tooth locates at basal 1/3, triangle-like, proceeded by more than 5 inner small teeth.  **Medium males** (42–47. 9mm): strongly incurved at basal 1/3, usually straight at apex; Apical teeth bifurcated, upper branch teeth usually equal or smaller than lower branch teeth; major mandibular tooth locates at basal 1/3, triangle-like, proceeded by 4 or 5 inner small teeth.  **Minor males** (< 42 mm): weakly incurved at basal 1/3, straight at apex; Apical teeth bifurcated, upper branch teeth usually equal or smaller with lower branch teeth; major mandibular tooth locates at basal 1/3, triangle-like, proceeded by less than 4 inner small teeth. | Reddish or brownish, narrower, or equal to head’s width. Clearly punctate; sometimes covered with one layer of small, yellowish setae. | Protibia usually with 3 or 4 small spines, apical tooth undeveloped, merely bifurcated; Mesotibia usually with 2 or 3 small, distinct spines; Mesotibia merely has 1 spine. Tibiae are usually reddish or brownish, femurs coved with remarkable yellowish stripes. | Shiny, brownish or merely reddish. Smooth and without any yellowish setae. |
| *Lucanus wuyishanensis*  *Lucanus liuyei* | 28.3–51.2 mm  28.6–52.3 mm | covered with a layer of short, yellowish amber setae; surface color brownish to reddish, shining metallic. | **Major males** (40–42.6 mm): mandible weekly incurved at basal 1/3, straight at the middle then strongly incurved at apical 1/4; the major inner mandibular tooth locates 2/3 of the apex, sharp, triangular protruding forward and inflated on both sides, 2 separated small inner mandibular teeth attached below the major inner mandibular tooth, 4 or 5 unclear, minor inner mandibular teeth continuously located on 1/2 of basal mandible; 4 small inner mandibular teeth densely distributed between the major tooth and the apical fork.  **Medium males** (35–39 mm): major inner mandibular tooth somewhat triangular, weekly inflated on both sides; > 4 unclear, minor inner mandibular teeth continuously located on 1/2 of the basal mandible, and > 4 small inner mandibular teeth densely distributed between the major tooth and the apical fork  **Minor males** (< 35 mm): major inner mandibular tooth weekly developed, single-point and not triangular; < 2 separated small inner mandibular teeth attached below the major inner mandibular tooth; > 3 unclear, minor inner mandibular teeth continuously located on 1/2 of basal mandible; > 2 small inner mandibular teeth densely distributed between the major tooth and the apical fork. | reddish to brownish, always the same color with the head; | protibia yellowish amber, bears with 5 markable spines from the mesotibia yellowish amber, with 2 undeveloped spines; metatibia yellowish amber, smooth and without any spines | reddish to brownish, color usually little brighter than head & pronotum; oval-shape, widest at the apical 1/4, strongly narrow at basal. |
