# Supplementary material for: ﻿Multilocus phylogeny and species delimitation suggest synonymies of two Lucanus Scopoli, 1763 (Coleoptera, Lucanidae) species names
Source: Zookeys. 2022 Dec 14;1135:139–55. doi: 10.3897/zookeys.1135.89257 (PMC9836572; doi:10.3897/zookeys.1135.89257)
Supplement: Supplementary material 3 — The mean genetic distance among populations in each collection area (K2P-distances) [file zookeys-1135-139_article-89257__-s003.docx]

|  | **1** | **2** | **3** | **4** | **5** | **6** | **7** | **8** | **9** | **10** | **11** | **12** | **13** |
| --- | --- | --- | --- | --- | --- | --- | --- | --- | --- | --- | --- | --- | --- |
| ***L, liuyei GX*** |  |  |  |  |  |  |  |  |  |  |  |  |  |
| ***L, liuyei GZ*** | **0.00676** |  |  |  |  |  |  |  |  |  |  |  |  |
| ***L, liuyei HN*** | **0.00819** | **0.01074** |  |  |  |  |  |  |  |  |  |  |  |
| ***L. wuyishanensis FJ*** | **0.00894** | **0.00872** | **0.01048** |  |  |  |  |  |  |  |  |  |  |
| ***L. wuyishanensis ZJ*** | **0.01066** | **0.01103** | **0.01075** | **0.00892** |  |  |  |  |  |  |  |  |  |
| ***L. wuyishanensis JX*** | **0.00824** | **0.00990** | **0.00654** | **0.00906** | **0.00970** |  |  |  |  |  |  |  |  |
| ***L. continnetalis FU,ZJ*** | **0.19603** | **0.19587** | **0.19268** | **0.19336** | **0.19142** | **0.19195** |  |  |  |  |  |  |  |
| ***L. swinhoei TW*** | **0.19123** | **0.19148** | **0.18890** | **0.18769** | **0.18583** | **0.18798** | **0.00729** |  |  |  |  |  |  |
| ***L. fujianensis GD*** | **0.21279** | **0.21604** | **0.21021** | **0.21553** | **0.21432** | **0.21225** | **0.16175** | **0.15920** |  |  |  |  |  |
| ***L. klapperichi*** | **0.19448** | **0.19537** | **0.19409** | **0.19754** | **0.19863** | **0.19710** | **0.19258** | **0.18722** | **0.19290** |  |  |  |  |
| ***L. fryi*** | **0.20583** | **0.20571** | **0.20897** | **0.20809** | **0.21122** | **0.20705** | **0.23211** | **0.22808** | **0.18857** | **0.19557** |  |  |  |
| ***L. smithii*** | **0.22586** | **0.22491** | **0.22299** | **0.22543** | **0.22458** | **0.22315** | **0.21506** | **0.21769** | **0.22306** | **0.24347** | **0.19622** |  |  |
| ***L. parryi*** | **0.22737** | **0.23028** | **0.22618** | **0.23121** | **0.23254** | **0.22808** | **0.21936** | **0.21649** | **0.20547** | **0.18402** | **0.17748** | **0.19871** |  |

**Supplementary file 3: Table S3:** The mean genetic distance among populations in each collection area (K2P-distances).
